# Long Non-Coding RNA and mRNA Profiling in Early-Stage Bovine Embryos Treated with Glutathione

**DOI:** 10.3390/antiox9050402

**Published:** 2020-05-08

**Authors:** Qinqin Guo, Lixin Cui, Weijun Sun, Feng Li, Haisheng Hao, Xueming Zhao, Huabin Zhu, Weihua Du

**Affiliations:** 1 Embryo Biotechnology and Reproduction Laboratory, Institute of Animal Science, Chinese Academy of Agricultural Sciences, Beijing 100193, China; b20173020101@cau.edu.cn (Q.G.); hshclx@163.com (L.C.); sunweijun@lybaybio.com (W.S.); feng-l@sinogene.com.cn (F.L.); haohaisheng@caas.cn (H.H.); zhaoxueming@caas.cn (X.Z.); zhuhuabin@caas.cn (H.Z.); 2 State Key Laboratory of Agrobiotechnology, College of Biological Sciences, China Agricultural University, Beijing 100193, China

**Keywords:** glutathione, embryo fertilized in vitro, mRNA transcriptomics, long noncoding RNA, glutathione metabolism

## Abstract

We measured differential expression profiles of genes and long non-coding RNA (lncRNA) using RNA sequencing in bovine embryos with or without glutathione (GSH) treatment. Bovine embryos fertilized in vitro were treated with GSH to blastocyst. Embryos at the 8-16-cell and morula stages were collected, with embryos without GSH treatment as the control. RNA was isolated, amplified, and sequenced. Differentially expressed genes (DEGs) and lncRNAs (DElncRNAs) were identified and bioinformatic analyses carried out. Transcript levels were confirmed using quantitative RT-PCR. A total of 4100 DEGs were identified, of which 3952 were in GSH-treated morulae and 884 in untreated morulae. More gene ontology (GO) terms were associated with GSH treatment than with control conditions. KEGG analysis showed that glutathione metabolism, citrate cycle, and metabolic pathways involving glycine, serine, and threonine were observed only in GSH-treated embryos. Among 4273 DElncRNAs identified, 59 were potentially important in GSH-treated embryo development, including 14 involved in glutathione metabolism. The 59 DElncRNAs co-expressed with protein-coding mRNAs involved similar GO terms and pathways as the DEGs. This appears to be the first comprehensive profiling of DEGs and DElncRNAs in bovine embryos fertilized in vitro with or without GSH, and the first systematic screen of potential lncRNAs in bovine embryos.

## 1. Introduction

Mammalian pre-implantation embryonic development is a comprehensive and finely controlled process that includes fertilization, cleavage divisions, genome activation, compaction, and blastulation. Following fertilization, the maternal-to-zygotic transition is the first critical transition in development. Following this, the developmental program is directed by the activated embryonic genome, instead of maternal proteins and mRNA [1]. Then, the transition from the 8-16-cell stage to the morula stage is vital for embryonic development [2], at which point the embryonic genome is activated, the blastomere compacts, and cleavage divisions occur [3]. Correct embryonic development requires expression of the appropriate genes and long non-coding RNAs (lncRNAs) [4].

Embryogenesis is easily affected by external factors such as temperature, carbon dioxide concentrations, and oxidative stress [5]. Oxidative stress, mediated by reactive oxygen species, can shift the intracellular redox balance toward oxidation [6,7]. This alters the cell fate determination, especially in embryos cultured or produced in vitro [8]. To combat such stress during in vitro maturation of oocytes or in vitro culture of embryos, antioxidants are often supplemented in the culture medium, and these include beta-mercaptoethanol [9], cysteine [10], taurine [11], vitamins C and E [12], l-carnitine [13], melatonin [14], and glutathione (GSH) [15,16].

GSH is a relatively small molecule that is ubiquitous in living organisms. It consists of three amino acids: glutamate (Glu), cysteine (Cys), and glycine (Gly). GSH can protect cells from oxidative damage by maintaining an intracellular redox state [17,18]. During embryonic development, GSH also mediates numerous processes by reversibly oxidizing Cys residues in various protein targets [19]. Adding GSH to culture medium increased the developmental rate of bovine morula and blastocyst produced by in vitro fertilization by 13% and 20%, respectively [20]. Our previous work showed that treating in vitro fertilized embryos with GSH significantly increased the rate of morula formation but not of 8-16-cell stage embryos [20]. How GSH achieves these effects is unclear. Elucidating the effects of GSH on embryonic development is essential not only for understanding mammalian reproductive biology but also for optimizing the outcomes of in vitro fertilization.

The present study is the first to directly compare gene and lncRNA expression between embryos cultured with or without GSH in order to reveal changes in developmental processes at the level of the entire genome. We focused on in vitro fertilized bovine embryos at the 8-16-cell and morula stages based on our previous work [20]. The results obtained here with bovine embryos may provide some clues about human embryos because of the similarities in their transcriptomes [2].

## 2. Materials and Methods

### 2.1. Animals and Ethics Statement

Holstein bulls (*n* = 3) ranging in age between 15 and 18 months were housed at the Shandong Dairy Cattle Center (Jinan, China). They were allowed free access to food and water. Semen was collected from bulls using an artificial vagina. A total of 24 ejaculates were collected and pulled together over 4 weeks. The semen was aliquoted and frozen in 0.25 mL straws.

All animal procedures were performed in line with the guidelines from the China Council on Animal Care, and all experimental protocols followed the requirements of the Institutional Animal Care and Use Committee of the Chinese Academy of Agricultural Sciences (permit IAS18028).

### 2.2. Reagents

All the chemicals used in this study were purchased from Sigma Chemicals (St. Louis, MO, USA) unless otherwise indicated.

### 2.3. Production and Collection of In Vitro Fertilized Bovine Embryos

Ovaries from an abattoir were transported at 37 °C to the laboratory. Cumulus-oocyte complexes were matured in TCM199 medium (GIBCO, Invitrogen, Carlsbad, CA, USA) supplemented with 10% fetal bovine serum (FBS; GIBCO, Invitrogen), 0.01 IU/mL follicle-stimulating hormone, 10 IU/mL luteinizing hormone, and 1 μg/mL estradiol for 22 h in an incubator at 38.5 °C in 5% CO_2_ and 100% relative humidity. Frozen semen in a straw of 0.25 mL was thawed at 37 °C and washed twice by centrifugation at 500 *g* for 8 min in Brackett–Oliphant medium [21] containing 10 μg/mL heparin, 10 mM caffeine, and 4 mg/mL bovine serum albumin (BSA). For insemination, oocytes were cultured with semen at 2 × 10^6^/mL in Brackett–Oliphant medium containing 4 mg/mL BSA and 10 μg/mL heparin.

After 8 h, the presumptive zygotes were cultured in vitro in modified-CRaa medium (mCRaa; 109.5 mM NaCl, 3.1 mM KCl, 26.2 mM NaHCO_3_, 0.8 mM MgCl_2_·6H_2_O, 1.19 mM KH_2_PO_3_, 0.4 mM Na pyruvate, 1.5 mM glucose, 0.55 mM l-lactate, 2% essential amino acid(EAA), 1% non-essential amino acid (NEAA), and 0.5% phenol red) supplemented with 6 mg/mL BSA for the early stage (0–48 h) or 10% FBS (GIBCO, Invitrogen) for the later stage (48–168 h). GSH was added at a 3 mM concentration to the culture medium of embryo from the beginning of culture in vitro to the blastocyst stage for 7 days [20]. Embryos at the 8-16-cell stage were collected at 72 h, and embryos at the morula stage were collected at 132 h. Embryos were treated with 1 mM protease to remove the zona pellucida and stored in RNAlater (Ambion, Thermo Fisher Scientific, Waltham, MA, USA) in liquid nitrogen for RNA-Seq and quantitative PCR.

### 2.4. RNA Isolation, Linear Amplification, Library Preparation, and Sequencing

RNA degradation and contamination were assessed on 1% agarose gels. RNA purity was checked using the NanoPhotometer spectrophotometer (Implen, Munchen, Germany). RNA concentration was measured using the Qubit RNA Assay Kit in a Qubit 2.0 Fluorometer (Life Technologies, Grand Island, NY, USA), and RNA integrity was assessed using the RNA Nano 6000 assay kit on a Bioanalyzer 2100 system (Agilent Technologies, Santa Clara, CA, USA). The 8-16-cell embryos and morula cultured with GSH in media were as the GSH treated group, while those at the same stages were cultured without GSH in media as control group, with two replicates per group.

RNA was prepared from pools of 20 embryos from each group. Cell lysis and first-strand cDNA synthesis were carried out using the SMARTer Ultra-Low RNA kit (Clontech Laboratories, Fremont, CA, USA) for Illumina sequencing. Briefly, First-strand cDNA was synthesized using 3’SMAT CDS primer, SMARTScribe^TM^ Reverse Transcriptase (RNase H minus) and SMARTer IIA Oligonucleotide. Then the first-strand cDNA was purified with SPRI beads and the double-strand cDNA was amplified using Advantage 2 polymerase Mixand IS PCR primer. Purification of double-strand cDNA was carried out with SPRI beads. After validation, cDNA was sheared. DNA fragments were adenylated on the 3’ end and ligated to NEBNext adaptors. In order to preferentially select cDNA fragments 150–200 bp in length, library fragments were purified with the AMPure XP system (Beckman Coulter, Beverly, MA, USA). Then, 3 µL of USER Enzyme (New England Biolabs, Beverly, MA, USA) was added to size-selected, adaptor-ligated cDNA at 37 °C for 15 min, followed by 5 min at 95 °C. Next PCR was performed with the Phusion High-Fidelity DNA polymerase, universal PCR primers, and Index (X) Primer. Finally, PCR products were purified (AMPure XP system) and library quality was assessed on an Agilent Bioanalyzer 2100 system.

Clustering of index-coded samples was performed on a cBot Cluster Generation System with the TruSeq PE Cluster Kit v3-cBot-HS (Illumina, San Diego, CA, USA). After cluster generation, the library preparations were sequenced on an Illumina Hiseq platform and 100 bp paired-end reads were generated. The eight libraries were distributed into a flow cell for sequencing in a Hi-Seq2000 (Illumina). The raw data were uploaded to the GeneSifter software (Geospiza, Seattle, WA, USA) for alignment based on UMD3.1 genome assembly as reference genome.

### 2.5. Mapping, Assembly, and Gene Expression Analysis

After sequencing, raw data were first processed through in-house Perl scripts. Then reads containing adapter, poly-N and PCR primer contamination were removed from the raw data. Q20, Q30, and GC content of clean data were calculated. Reference genome and gene model annotation files were downloaded from the Ensemble Genome Browser [22]. The reference genome index was assembled using Bowtie v2.2.3 (http://bowtie-bio.sourceforge.net/bowtie2/index.shtml), and paired-end clean reads were mapped to the reference genome using TopHat v2.0.12 (http://ccb.jhu.edu/software/tophat/index.shtml). HTSeq v0.6.1 (https://htseq.readthedocs.io/en/master/) was used to count the number of reads mapped to each gene. The fragments per kilobase of transcript sequence per million fragments mapped (FPKM) of each transcript was calculated based on the length of the gene and reads mapped to this gene. FPKM is influenced by sequencing depth and gene length for read count and is currently the most commonly used method for estimating gene expression [23].

### 2.6. Novel Transcript Prediction and Alternative Splicing Analysis

The Cufflinks v2.1.1 (http://cole-trapnell-lab.github.io/cufflinks/) reference annotation-based transcript assembly method was used to construct and identify both known and novel transcripts from TopHat alignment results. Alternative splicing events were assigned to 12 basic types using Asprofile v1.0 (http://ccb.jhu.edu/software/ASprofile/). The number of alternative splicing events in each sample was estimated.

### 2.7. Identification of Putative lncRNAs

Each transcript was classified as either coding or non-coding using a stepwise method. All candidate transcripts were scored for their coding potential using PhyloCSF (GitHub Inc., San Francisco, CA, USA) [24] and the Coding Potential Assessment Tool (CPAT) v1.2.1 (25). PhyloCSF uses a multispecies nucleotide sequence alignment to identify conserved protein-coding regions based on a statistical comparison of phylogenetic codon models. We used a five-species alignment of cow, human (hg19), mouse (mm9), rat (rn4), and dog (CanFam2). Pairwise alignments were obtained from the University of California-Santa Cruz Genomics Institute (Santa Cruz, CA, USA, http://genome.ucsc.edu). All transcripts which have a negative score were kept as potential non-coding candidates. CPAT was also applied to all candidate transcripts to provide a second, independent assessment of their coding potential [25,26]. All candidate transcripts were translated in silico into the three possible open reading frames using a custom script and compared against the Pfam protein families database v27.0 [27] using the hmmscan algorithm in HMMER3 v3.1b1 (European Bioinformatics Institute, Hinxton, Cambridgeshire, UK). Candidate transcripts with known protein motifs were discarded.

### 2.8. Analysis of Differential Expression

Differentially expressed genes (DEGs) and differentially expressed lncRNAs (DElncRNAs) between embryos treated or not with GSH were identified using DESeq R v1.18.0 [28]. For each time point of each treatment condition, 2 independent pooled samples (*n* = 20 embryos for each) were used for analysis. DESeq assesses the statistical significance of differential expression using a model based on a negative binomial distribution. P values were adjusted using Benjamini and Hochberg’s false discovery rate method [29]. Differential expression was considered significant when the corrected *p* was smaller than 0.05, and the log 2 (fold change) was at least 1.

### 2.9. GO and KEGG Enrichment Analysis of DEGs

Gene ontology (GO) enrichment of DEGs, was analyzed using GOseq R [30] with correction for gene length bias. GO terms with corrected P values smaller than 0.05 were considered significantly enriched in DEGs. The KOBAS software was used to test the statistical enrichment in KEGG pathways (*p* < 0.07).

### 2.10. Prediction of Co-Expressed Genes and Neighborhood Genes of DElncRNAs

Co-expression between DElncRNAs and mRNAs was evaluated using Spearman’s correlation test [31]. Co-expression was considered significant when the correlation coefficient was greater than 0.7 or smaller than −0.7 and when *p* < 0.05. The network of DElncRNAs and mRNAs was constructed using Cytoscape v3.2.1 (http://www.cytoscape.org/release_notes_321.html). The protein-coding genes (PCGs) within about 300 kb upstream or downstream of lncRNAs in the genome were searched using the Ensemble Genome Browser [32]. These neighborhood genes were considered potential cis target genes. GO annotation and KEGG pathway enrichment analysis were performed for these co-expressing genes and the target genes of DElncRNA, respectively, to investigate the biological processes and signaling pathways that lncRNAs were mainly involved in and the functions of lncRNAs.

### 2.11. Quantitative Real-Time Polymerase Chain Reaction (RT-qPCR)

RNA-Seq was performed to validate 10 DEGs (ATP5L, PSMA3, UGP2, RPS3A, COX7A2, MGST1, IDH1, RRM2, OOSP1, and THAP9) and 8 lncRNAs (CUFF.33095.2, CUFF.52291.1, CUFF.55358.1, CUFF.152963.1, CUFF.17837.1, CUFF.21976.3, CUFF.42178.2, and CUFF.91156.1). Expression levels were measured at the 8-16-cell and morula stages using the Power SYBR^®^ Green Cells-to-Ct™ kit (Life Technologies) and the primers listed in Table S1. All experiments were performed at least three times. Relative expression was calculated using the 2-ΔΔCT method and normalized to the expression of glyceraldehyde-3-phosphate dehydrogenase (*GAPDH*) in each sample. To calculate fold change in expression of DEGs and lncRNAs, the levels at the morula stage were divided by the levels at the 8-16-cell stage.

## 3. Results

### 3.1. Expression Profiling of In Vitro-Fertilized Bovine Embryos

**Figure 1:**
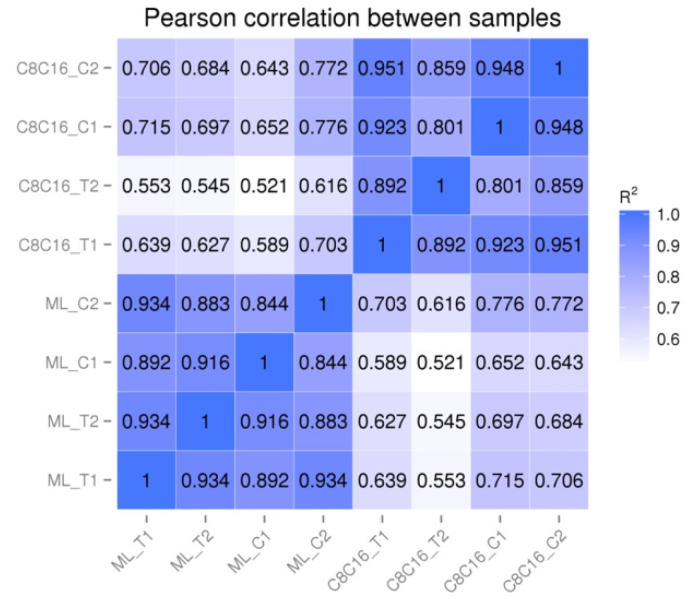
Heat map of bovine embryo biological replicates at the 8-16-cell or morula stage. Duplicate in vitro-fertilized bovine embryos were treated with GSH or not for 7 days, then lysed at the 8-16-cell or morula stage for RNA extraction and sequencing. The Pearson correlation coefficient was used to measure reproducibility of sample preparation and sequencing results. The blue to white color spectrum represents correlation coefficients ranging from 1 to 0.553, indicating high to low correlations. Abbreviations: GSH, glutathione; C8C16_C1, 8-16-cell Control #1; C8C16_C2, 8-16-cell Control #2; C8C16_T1, 8-16-cell + GSH #1; C8C16_T2, 8-16-cell + GSH #2; ML_C1, Morula Control #1; ML_C2, Morula Control #2; ML_T1, Morula +GSH #1; ML_T2, Morula +GSH #2.

Eight RNA-seq libraries were constructed from embryos at the 8-16-cell and morula stages that had been treated or not with GSH. In total, 314,135,947 sequencing reads from duplicate samples were obtained (Table S2). The Pearson correlation coefficients of biological replicates showed the reproducibility of sample preparation and sequencing technology (Figure 1 and Table S3). The total number of detectable genes (FPKM > 0.5) was 13,498 in 8-16-cell embryos and 11,527 in morula embryos (Table S4), corresponding to about 50% of the estimated 22,000 genes in the bovine genome.

### 3.2. DEG Analysis during Development from the 8-16-Cell Stage to Morula Stage in GSH-Treated and Untreated Embryos

**Figure 2:**
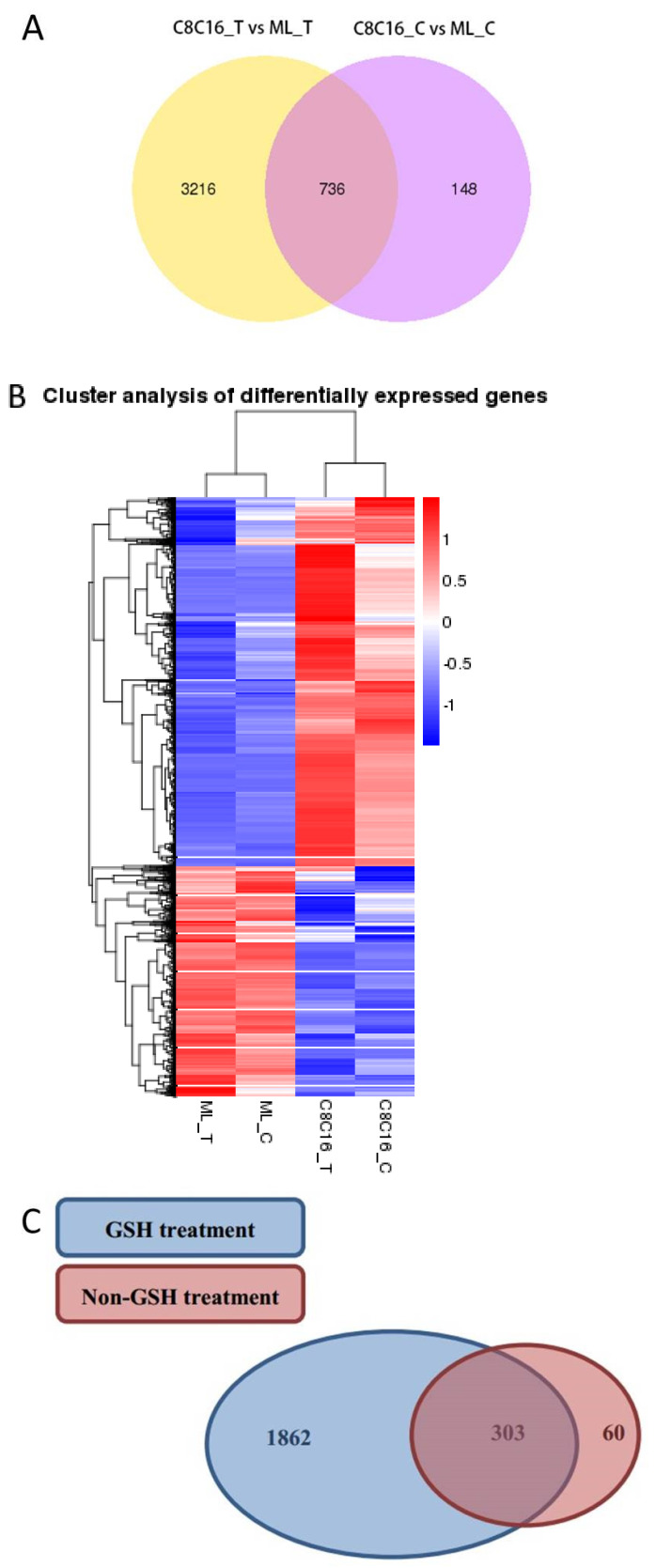

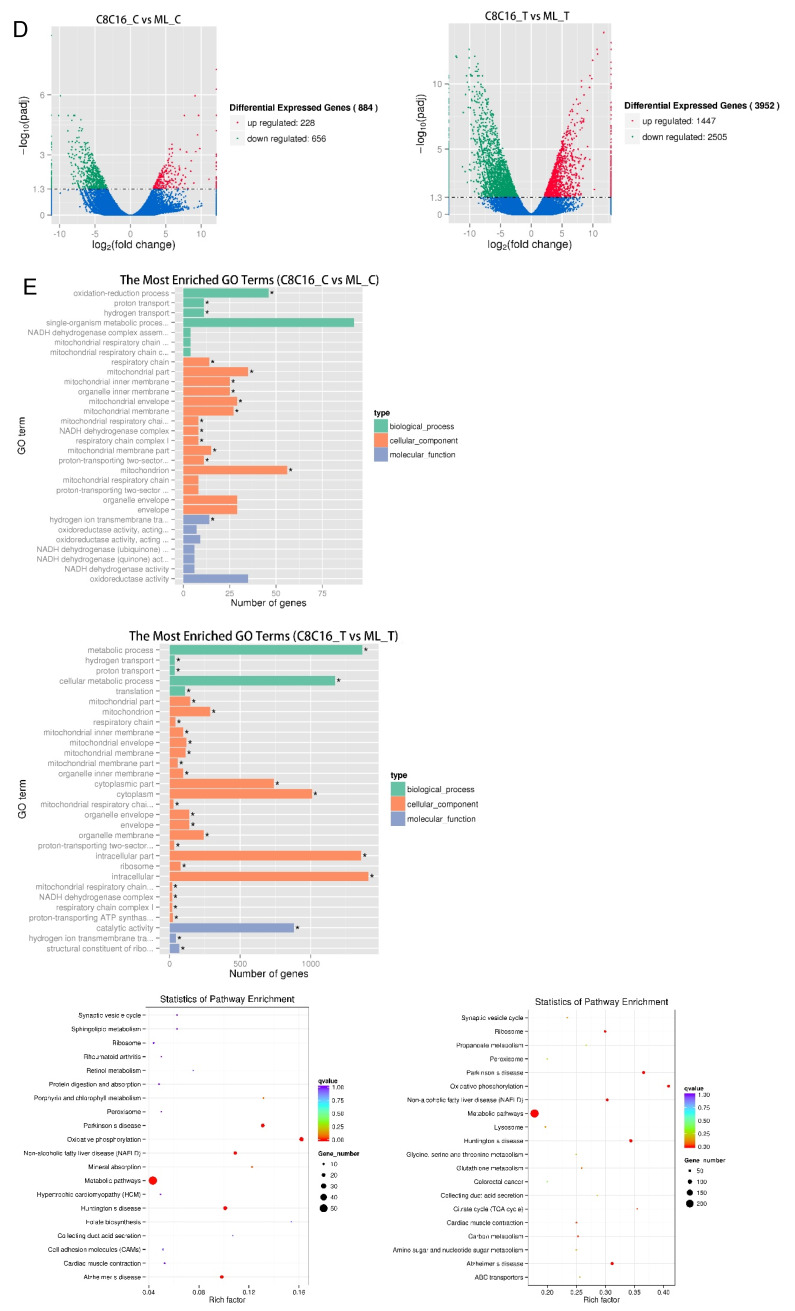
Differentially expressed genes (DEGs) during embryo development from the 8-16-cell stage to morula with or without glutathione treatment. (**A**) Venn diagram of DEGs between the two groups. The yellow area represents the DEGs in the GSH-treated embryos; the purple area, DEGs in the untreated embryos; and the overlapping area, DEGs common between the two groups of embryos. (**B**) Hierarchical clustering of DEGs between the 8-16-cell stage embryos and morula with or without GSH treatment. The color spectrum indicates normalized levels of gene expression. (**C**) Venn diagram of unique DEGs in each group of embryos. The blue area represents unique DEGs in GSH-treated embryos; the orange area, unique DEGs in the untreated group; and the overlapping area, unique DEGs common to the two groups. (**D**) Volcano plots showing fold change of up- or down-regulated genes or unchanged (blue) genes between the untreated (left) and GSH-treated (right) groups. (**E**) GO analysis (top row) and KEGG analysis (bottom row) of DEGs during embryo development from the 8-16-cell stage to morula with or without GSH treatment.

In embryos not treated with GSH, we identified 884 genes (363 known and 521 new) that were differentially expressed between the 8-16-cell and morula stages (Figure 2A and Table 1). In embryos that were treated with GSH, we identified 3952 genes (2165 known and 1787 new) that were differentially expressed between the two developmental stages. Hierarchical clustering of the DEGs showed two gene clusters for the two developmental stages (Figure 2B). Among the unique DEGs, 60 were expressed only in untreated embryos, 1862 only in GSH-treated embryos, and 303 in both. We speculate that these 303 genes are necessary for 8-16-cell embryos to develop to the morula stage (Figure 2C). In the progression from the 8-16-cell stage to morula stage, 193 genes were down-regulated and 170 up-regulated in untreated embryos, compared to 1148 genes down-regulated and 1017 up-regulated in GSH-treated embryos (Figure 2D, Tables S5 and S6). These results suggest that GSH activates protein synthesis, cell division progression, and blastomere differentiation.

DEGs in untreated embryos were enriched for 397 GO terms related to oxidation–reduction, ATP biosynthesis, and the oxidative respiratory chain (Table S5). DEGs in GSH-treated embryos were enriched for those terms as well as DNA synthesis, translation, and metabolism, with total of 651 GO terms (Figure 2E and Table S6). KEGG pathway analysis revealed that 22 pathways were enriched in GSH-treated embryos, while 12 pathways were enriched in untreated embryos. These pathways included GSH metabolism; citrate cycle (TCA cycle); glycine, serine, and threonine metabolism, as well as vitamin metabolism and absorption pathways. These pathways aligned well with the GO enrichment (Figure 2E, Tables S5 and S6). It appears that GSH treatment significantly affected expression of genes in the GSH metabolism pathway (map00480) during the transition from the 8-16-cell stage to the morula stage.

### 3.3. DEG Analysis between GSH-Treated and Untreated Embryos within Each Developmental Stage

The results above suggest that the expression of many genes changes during the transition from the 8-16-cell to morula stage, regardless of whether GSH is used. In contrast, when we compared GSH-treated embryos and untreated embryos at the same developmental stage, we found negligible differences. Only three DEGs were observed between the two types of embryos at the 8-16-cell stage, while no DEGs were detected between the two types at the morula stage (Table S7).

### 3.4. Identification of lncRNAs of In Vitro-Fertilized Bovine Embryos

**Figure 3:**
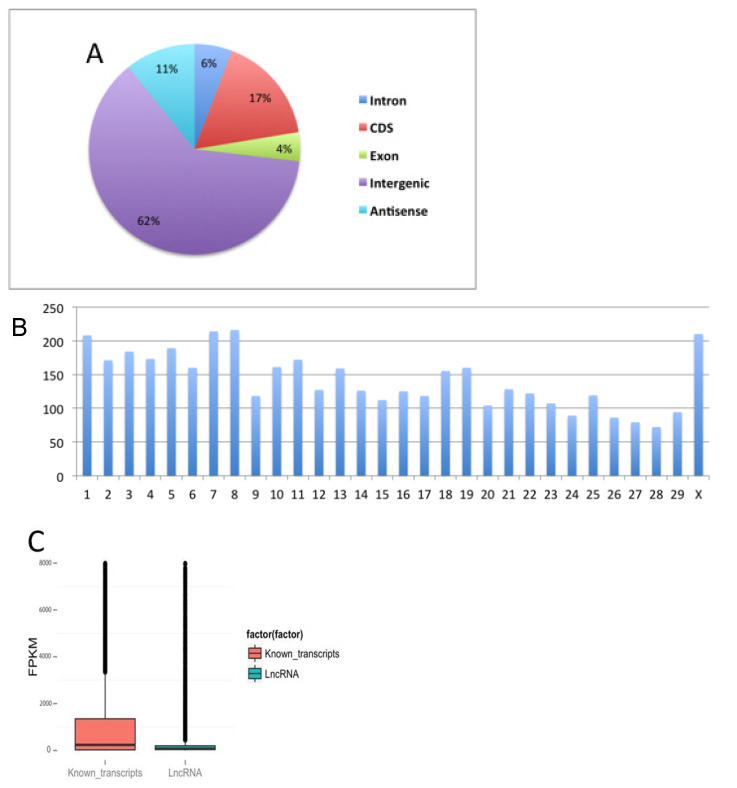

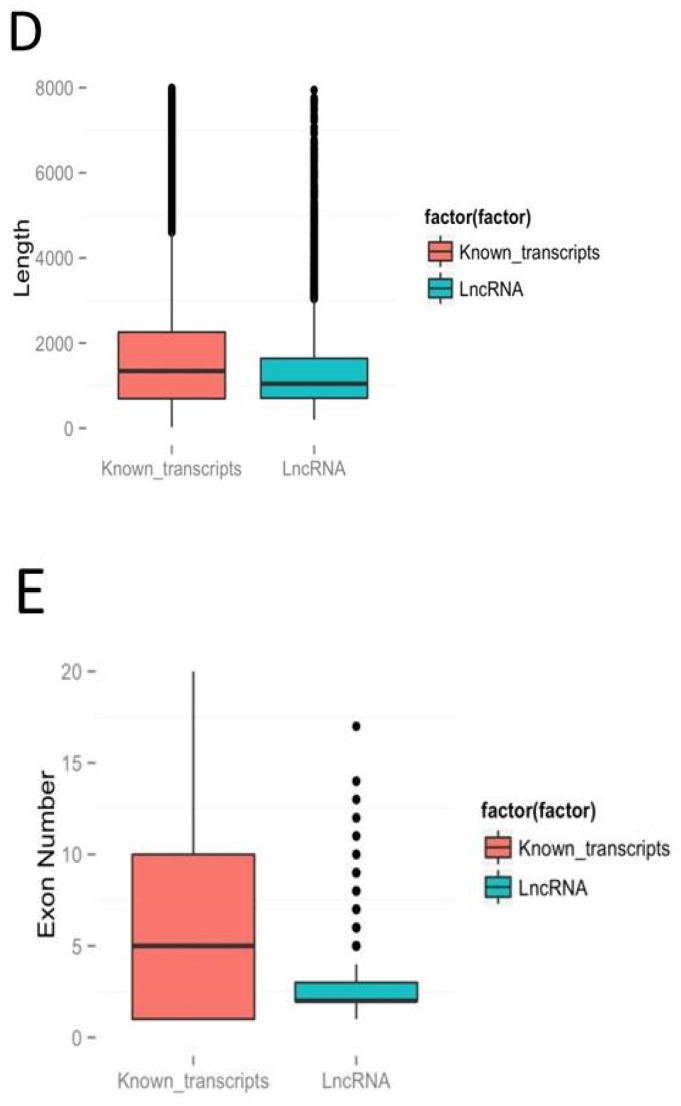
Location, chromosomal distribution and characterization of lncRNAs in bovine embryos at the 8-16-cell and morula stages with or without glutathione treatment. (**A**) Location of lncRNAs within genetic sequences. (**B**) Distribution of lncRNAs in bovine chromosomes. (**C**) Comparison of expression levels of lncRNAs with known transcripts. The mean transcript expression level (FPKM) of lncRNAs. (**D**) Length comparison between lncRNAs and known transcripts. (**E**) Comparison of the number of exons between lncRNAs and known transcripts. Abbreviations: GSH, glutathione; CDS, coding sequence; FPKM, per million fragments mapped; lncRNA, long non-coding RNA.

Of the 4273 predicted lncRNAs, 62% came from intergenic regions (Figure 3A and Table S8). The results showed no chromosomal bias of the lncRNAs, suggesting that we achieved genome-wide coverage (Figure 3B). The FPKM of predicted lncRNAs was 2.17, compared to 378.925 for known transcripts (Figure 3C). The average length of lncRNAs was 1368.69 bp, which was shorter than the 1708.6 bp for known transcripts (Figure 3D). The average numbers of exons were 2.52 in lncRNAs and 8.44 in known transcripts (Figure 3E). These characteristics are consistent with previously identified lncRNAs [33,34], and suggest that our lncRNA predictions are reliable.

### 3.5. DElncRNA Analysis during Development from the 8-16-Cell Stage to Morula Stage in GSH-Treated and Untreated Embryos

**Figure 4:**
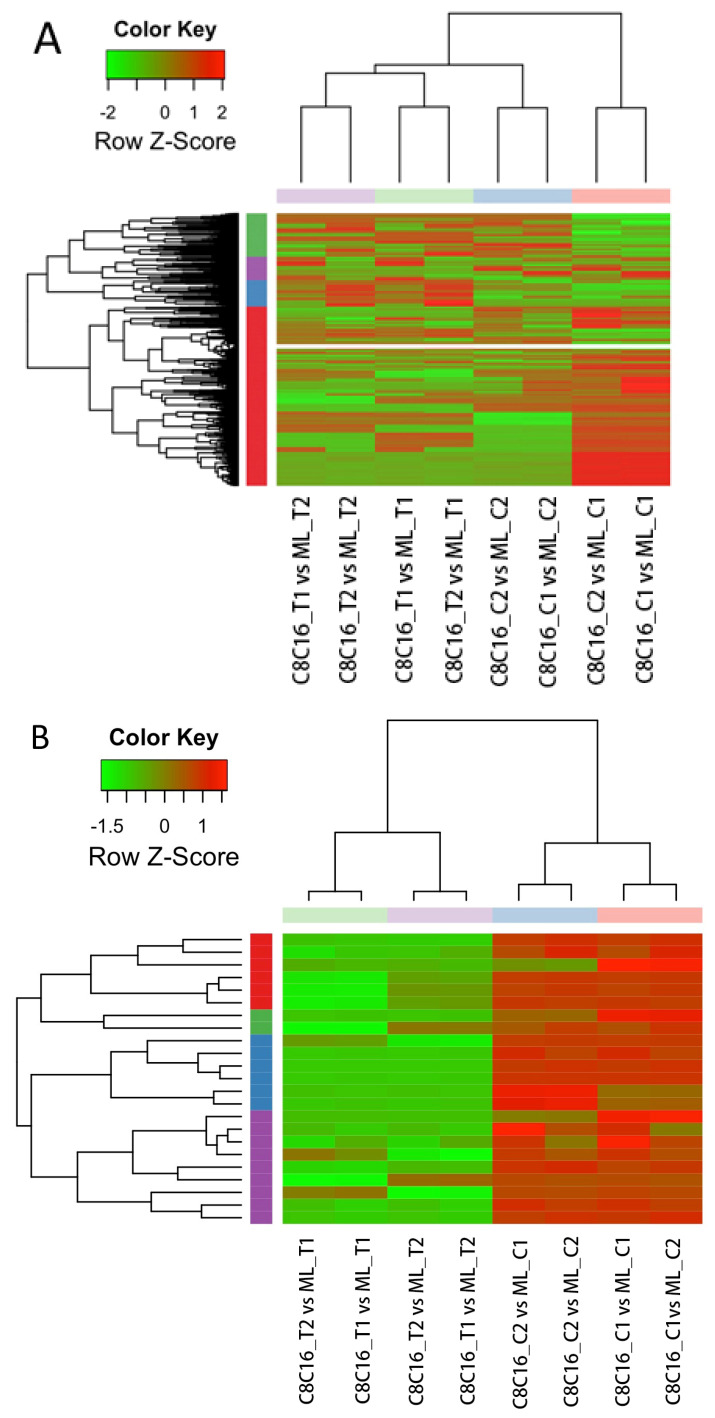

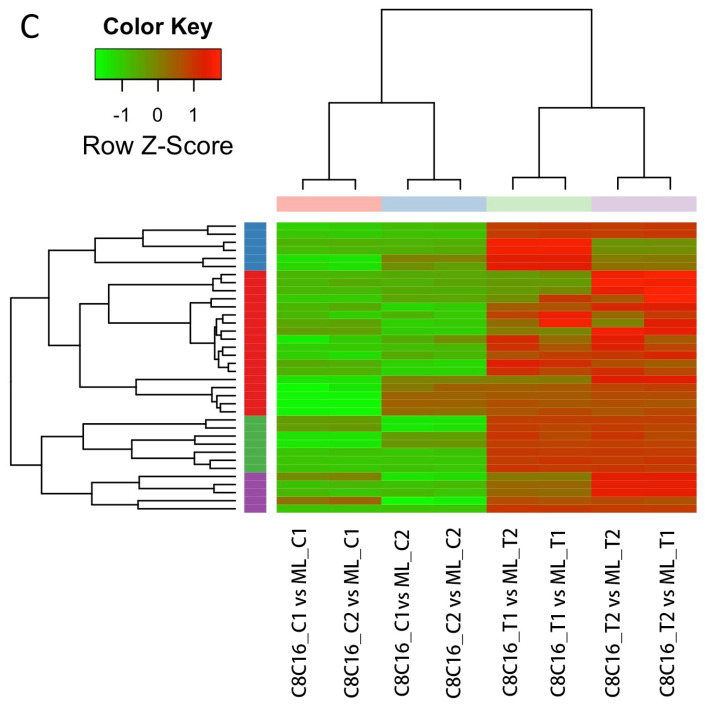
Heat map of hierarchical clustering for lncRNAs. (**A**). Heat map of 4273 predicted lncRNAs. Two biological replicates at the 8-16-cell stage or morula stage are presented together. (**B**) Heat map of 23 lncRNAs up-regulated (red) in the control group and down-regulated (green) in GSH-treated embryos. (**C**) Heat map of 36 lncRNAs down-regulated (green) in untreated embryos and up-regulated (red) in GSH-treated embryos.

In untreated embryos, we identified 4091 lncRNAs that were differentially expressed between the 8-16-cell and morula stages (1400 up-regulated and 2691 down-regulated) (Table 1). In GSH-treated embryos, we identified 4104 lncRNAs (1367 up-regulated and 2737 down-regulated). When we examined only embryos in the 8-16-cell stage, we identified 3973 lncRNAs that were differentially expressed in the presence of GSH (2245 up-regulated and 1728 down-regulated). When we examined only embryos in the morula stage, 3528 DElncRNAs were differentially expressed in the presence of GSH (1427 up-regulated and 2101 down-regulated). Data were similar for biological replicates (Figure 4A).

Remarkably, of the total of 4273 predicted lncRNAs, 23 were up-regulated in untreated morulae compared with untreated 8-16-cell stage embryos, but down-regulated in GSH-treated morulae compared with treated 8-16-cell stage embryos (Figure 4B and Table S9), and another 36 were down-regulated in untreated morulae compared with untreated 8-16-cell stage embryos, but up-regulated in GSH-treated morulae compared with treated 8-16-cell stage embryos (Figure 4C and Table S9). The top five lncRNAs that were up-regulated with highest fold change by GSH treatment included CUFF.17837.1, CUFF.21976.3, CUFF.30537.1, CUFF.50309.1, and CUFF.87588.1.

### 3.6. Co-Expression Network Analysis of DElncRNAs and mRNAs

Co-expression analysis is commonly used to predict the mechanisms of lncRNAs. We took a set of 59 DElncRNAs (as mentioned in previous paragraph) and searched for co-expressed protein-coding mRNAs from 884 DEGs and 3952 DEGs identified in the untreated and GSH-treated embryos.

**Figure 5:**
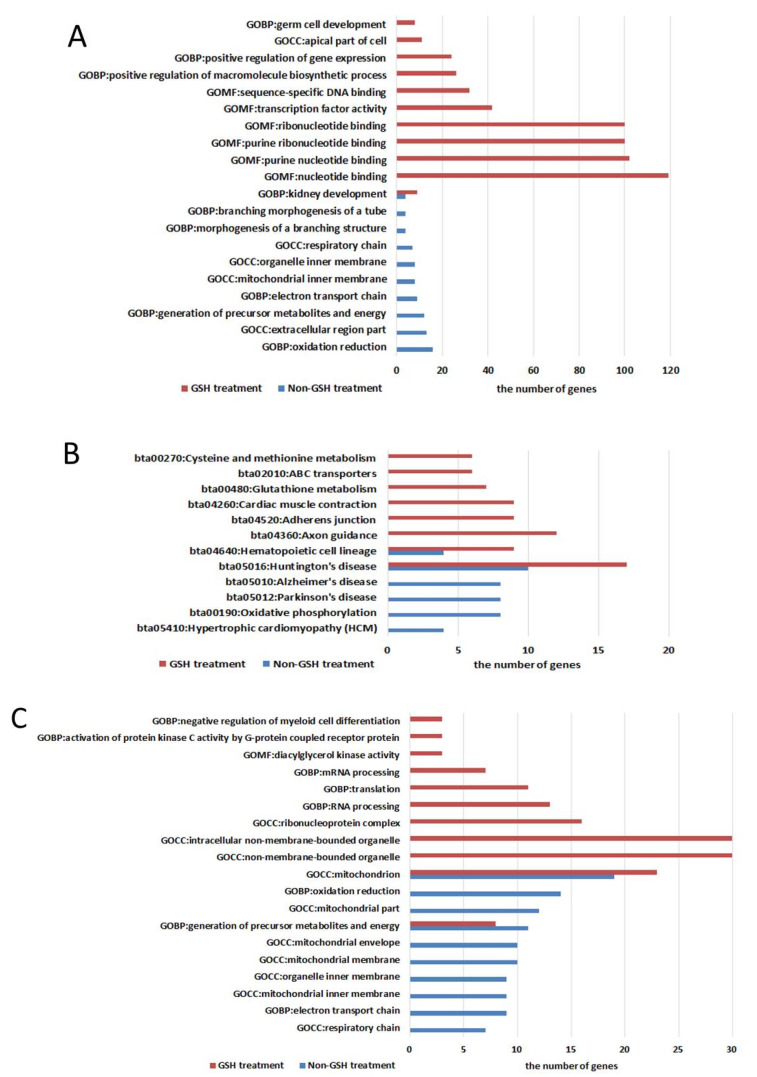

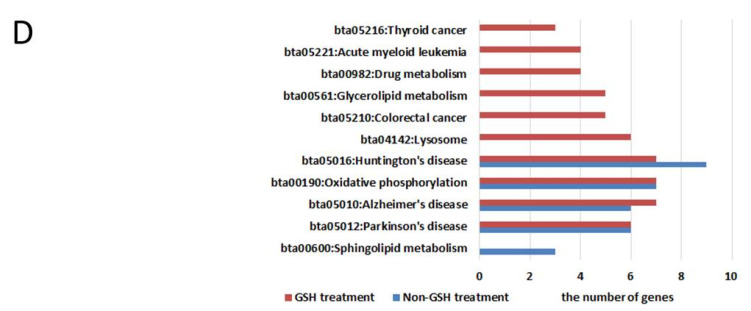
Functional analysis of protein-coding mRNAs co-expressing with differentially expressed lncRNAs. (**A**) GO and (**B**) KEGG pathway analysis output of protein-coding mRNAs co-expressing with 23 DElncRNAs in untreated embryos (C, blue) and GSH-treated embryos (T, red) groups. (**C**) GO and (**D**) KEGG pathway analysis output of protein-coding mRNAs co-expressing with 36 DElncRNAs in untreated embryos (C, blue) and GSH-treated embryos (T, red).

In untreated embryos, 23 DElncRNAs were up-regulated at morula stage and co-expressed with 599 mRNAs while they were down-regulated in GSH-treated morulae and co-expressed with 2554 mRNAs (Figure 5, Tables S10 and S11). The associated GO terms enriched by those mRNAs in untreated and GSH-treated embryos involved commonly kidney development, urogenital system development, sexual reproduction, proteinaceous extracellular matrix, cell migration, cell motility and localization of cell. KEGG analysis revealed that mRNAs co-expressing with 23 DElncRNAs were enriched commonly in two pathways: Huntington’s disease and Hematopoietic cell lineage in untreated and GSH-treated embryos. Glutathione metabolism and cysteine and methionine metabolism were specific to embryos with GSH treatment.

In untreated embryos, 36 DElncRNAs were down-regulated at the morula stage and co-expressed with 483 mRNAs while they were up-regulated in GSH-treated morulae and co-expressed with 609 mRNAs (Figure 5, Tables S12 and S13). The GO terms enriched by those mRNAs in untreated and GSH-treated embryos involved commonly mitochondrion and generation of precursor metabolites and energy. KEGG analysis revealed that mRNAs co-expressing with 36 DElncRNAs were enriched commonly in four pathways: Huntington’s disease, Parkinson’s disease, Alzheimer’s disease, and oxidative phosphorylation in untreated and GSH-treated embryos. Sphingolipid metabolism was specific to untreated embryos.

According to the correlation coefficient analysis, a large number of DEGs was found to co-express with 59 DElncRNAs which has opposite change trend in untreated and GSH-treated embryos (Table 2). Importantly, the DElncRNA networks differed in the presence or absence of GSH at each time point, emphasizing the potentially extensive, pleiotropic effects of GSH on embryonic development.

### 3.7. Analysis of Protein-Coding Genes in the Proximity of DElncRNAs

The cis targets of many lncRNAs are genes lying nearest to them along the chromosome. We then conducted functional enrichment analysis for the genes nearest 300 kb cut-off for the 59 DElncRNAs. We identified 145 target genes in proximity to 23 DElncRNAs, and these genes were involved in 19 GO terms, primarily comprising biological regulation, metabolic processes, and membrane components. Only one pathway, cell cycle, was enriched (Table S14). Altogether 154 target genes were identified in proximity to 36 DElncRNAs and participated in five GO terms consisting of biological processes and molecular functions such as production of molecular mediator of immune response, immunoglobulin production, growth factor binding, inward rectifier potassium channel activity, and voltage-gated cation channel activity (Table S15).

### 3.8. Real-Time PCR Validation of DEGs and DElncRNAs in Embryos Treated with GSH or Not

To validate the throughput data from RNA-Seq, RT-qPCR was performed on 10 DEGs and 8 DElncRNAs at random in 8-16-cell and morula embryos treated with GSH (Table S16). Eight of the selected DEGs (ATP5L, PSMA3, UGP2, RPS3A, COX7A2, MGST1, IDH1, and RRM2) were up-regulated and two (OOSP1 and THAP9) were down-regulated between the two embryonic stages. Three of the DElncRNAs (CUFF.33095.2, CUFF.52291.1, and CUFF.55358.1) were down-regulated and five (CUFF.152963.1, CUFF.17837.1, CUFF.21976.3, CUFF.42178.2, and CUFF.91156.1) were up-regulated between the two embryonic stages. The fold changes for the 10 DEGs and 8 DElncRNAs were similar between RT-qPCR and RNA-Seq.

## 4. Discussion

As shown previously by our group, GSH supplementation in culture medium greatly improves the development of in vitro-fertilized bovine embryos [20], but how GSH or other antioxidants improve embryonic development in vitro is unclear. Here we report the first profiling of DEGs and DElncRNAs in bovine embryos fertilized in vitro with or without GSH treatment.

We found that four times more genes were differentially expressed between the 8-16-cell stage and morula stage in the presence of GSH than in its absence. This suggests that GSH stimulates extensive changes in gene expression during embryonic development. Paradoxically, the expression of only three genes differed between 8-16-cell embryos in the presence or absence of GSH, and no genes differed in expression at the morula stage. This may mean that GSH exerts greater effects on gene expression during the 8-16-cell stage. Future work should examine in more detail how the effects of GSH change during the complex, highly coordinated developmental pathway. Such work should also verify our results, since even small changes in the threshold values to define differential expression can lead to large changes in the numbers of DEGs and DElncRNAs.

Some of the DEGs that we identified in the presence of GSH belonged to the GSH metabolism pathway, similar to a previous study on zebrafish embryo [8]. However, most genes enriched in GSH metabolism were down-regulated. We speculate that exogenous GSH in culture medium leads to higher intracellular GSH levels, which down-regulate GSH metabolism genes. Furthermore, the expression of the inhibitor of DNA-binding 3 (ID3) was down-regulated during the development in GSH-treated embryos. The transcriptional regulator ID3 can promote cell apoptosis [35], so ID3 may be a cause of embryo death without GSH treatment after the morula stage; GSH may antagonize the effects of ID3, increasing the blastocyst rate [20].

In this study, the GO and KEGG pathway analysis of DEGs showed that GSH-treated embryos were more active than untreated ones in several biological processes such as DNA synthesis, protein synthesis, GSH metabolism, and material metabolism. Exogenous GSH also accelerated the division and differentiation of blastomeres. The underlying reason may be the changes induced by GSH in its own metabolic pathway. We found isocitrate dehydrogenase genes IDH1 and IDH2 to be enriched in the GSH metabolism pathway, TCA cycle, carbon metabolism, metabolic pathways, and peroxisome. Ribonuclease reductase modulator genes (RRM1 and RRM2B) and glutathione S-transferase mu 3 (GSTM3) are involved in both GSH metabolism and metabolic pathways. In addition, NADPH is involved in intracellular antioxidation and in redox reactions of GSH metabolism, nucleic acids, and lipid metabolism [36]. We hypothesize that the beneficial effects of GSH on embryo development reflect, in part, changes in the GSH metabolism pathway.

Furthermore, a comparison of DEGs was carried out between human and bovine embryos treated with GSH. According to the RNA-seq data of human embryo [37], they identified 3688 genes that differentially expressed between the 8-cell stage embryos and morulae. Compared with the 3952 DEGs identified in embryos treated with GSH at the two developmental stages in this study, there were more than 3500 different genes. Among those genes, we found 17 genes associated with embryo development such as SMAD family member 4 (*SMAD4*), GINS complex subunit 1 (*GINS1*), HNF1 homeobox B (*HNF1B*), etc. (Table S6). SMAD4 is a member of transforming growth factor ß (TGF ß) superfamily signaling and controls various aspects of female fertility. After in vitro maturation of porcine oocyte, the expression level of *SMAD4* gene was decreased significantly, which may be related to regulation of folliculogenesis and oogenesis [38]. *SMAD4* mRNA in bovine oocytes increased during in vitro maturation, peaked in 2-cell stage and 8-cell stage embryos, decreased at morulae, and remained low at blastocyst. Inhibiting SMAD4 expression in embryos, the developmental rates reduced. Therefore, SMAD4 is obligatory for early embryonic development of bovine embryos [39]. In this study, the levels of *SMAD4* mRNA were increased significantly in bovine morula treated with GSH, which may cause the increase of the morula rate. So that, high expression of *SMAD4* gene can be a potential marker for bovine and human embryos with good developmental competence.

There are tens of thousands of lncRNA genes in the mouse and human genomes and they have distinct functions [37,40], but lncRNAs in bovine embryos are still poorly identified and no systematic screening of potential lncRNAs has been reported. Previous studies showed that genes encoding lncRNAs are shorter in length, have shorter transcripts, and contain fewer exons than protein-coding genes and transcripts [41,42,43]. The lncRNAs identified in the present study have the same characteristics as those of a previous study on bovine muscle [26]. We also found that lncRNAs play very important roles in the process of embryonic development. According to our results, 4273 lncRNAs were predicted and their expression differed substantially between untreated and GSH-treated embryos. Those lncRNAs were clustered according to the developmental stage and the lncRNAs from embryos at 8-16-cell and morula stages were separated, whether the embryos had been treated with GSH or not. We focused on 59 DElncRNAs whose expression trends were contrary in untreated and GSH-treated embryos and their co-expression network was analyzed. The results showed that almost all GO terms contained more genes from GSH-treated embryos than from untreated embryos. Oxidation–reduction, respiratory chain, electron transport chain, and mitochondrial envelope occurred only in untreated embryos, while nucleotide binding, purine nucleotide binding, and germ cell development occurred only in GSH-treated embryos. Mitochondrion and generation of precursor metabolites and energy, and kidney development occurred commonly in both embryos. DEGs from both types of embryos occurred in pathways related to hematopoietic cell lineage, Huntington’s disease, Parkinson’s disease, oxidative phosphorylation, and Alzheimer’s disease. Sphingolipid metabolism was seen only in untreated embryos; lysosome, drug metabolism, colorectal cancer, acute myeloid leukemia, and thyroid cancer were seen only in GSH-treated embryos. In GSH-treated embryos, GSH metabolism was enriched among protein-coding mRNAs that co-expressed with 14 DElncRNAs, but this enrichment was not observed in untreated embryos (Table S17). This supports the idea that GSH profoundly affects gene expression in bovine embryos fertilized in vitro.

As reported in previous studies, the target genes of lncRNAs can be divided into cis targets located near the lncRNA, and trans targets that may be anywhere [44,45]. To predict trans targets, we analyzed co-expression of the 59 DElncRNAs with DEGs that we identified in untreated and GSH-treated embryos. The number of mRNAs co-expressing with DElncRNAs was three times higher in GSH-treated embryos than in untreated embryos. KEGG analysis showed that the protein-coding genes co-expressing with 14 lncRNAs participate in GSH metabolism, suggesting that these lncRNAs are particularly significant for embryonic development (Table S17). In the present study, 80% of lncRNAs were within 300 kb of the nearest gene and likely cis target, consistent with a previous report on human urothelial cancer [46]. As an example, many genes were in the vicinity of lncRNA CUFF.21976.3, including up-regulated ATP6V1C2, RRM2, and GRHL1 as well as down-regulated KLF11. Expression of these four genes changed significantly from the 8-16-cell stage to the morula stage in GSH-treated embryos, but not in untreated embryos. RRM2 plays a key role in GSH synthesis and metabolism pathway [47]. KLF11 (Kruppel-like factor 11) can induce cell death by down-regulating Bcl-X expression [48]. ATP6V1C2 and GRHL1 also play key roles in embryo development, cell cycle, and substance metabolism [49,50]. GSH treatment may up-regulate the expression of lncRNA CUFF.21976.3, leading to decreased expression of KLF11. This may increase cell viability and the rate of embryos that develop normally.

## 5. Conclusions

In conclusion, our results indicate that the expression of many genes and lncRNAs changes as embryos develop from the 8-16-cell stage to the morula stage after GSH treatment. The GO terms enriched for DEGs in GSH-treated embryos included DNA synthesis, translation, metabolism, and GSH metabolism. Among 4273 predicted lncRNAs in the bovine genome, 59 DElncRNA had different expressing trends in untreated and GSH-treated groups. Protein-coding mRNAs co-expressed with those 59 DElncRNAs involving similar GO terms and pathways as the DEGs. We further identified 14 DElncRNAs that participate in GSH metabolism and that may be particularly significant for embryonic development. Our results provide the first comprehensive profile of mRNA and lncRNAs in in vitro-fertilized bovine embryos treated with GSH.

## Figures and Tables

**Table 1:** Quantification of differentially expressed genes and lncRNAs in bovine embryos at 8-16-cell and morula stages.

Types	Change Trend	C8C16_C vs. ML_C	C8C16_T vs. ML_T	C8C16_C vs. C8C16_T	ML_T vs. ML_C
Genes	Up	228	1447	0	0
	Down	656	2505	3	0
	Total	884	3952	3	0
LncRNAs	Up	1400	1367	2245	1427
	Down	2691	2737	1728	2101
	Total	4091	4104	3973	3528

Abbreviations: C8C16_C, 8-16-cell Control; C8C16_T, 8-16-cell + GSH; ML_C, Morula Control; ML_T, Morula + GSH.

**Table 2:** Composition of differentially expressed lncRNAs networks with differentially expressed genes in untreated and treated embryos.

No. of DElncRNAs	Change Trend	Embryos	Nodes	Connections
23	up	untreated	623	4510
	down	treated	2578	18,345
36	down	untreated	517	1700
	up	treated	645	2845

Abbreviations: DElncRNAs, differentially expressed lncRNAs.

## Supplementary Materials

The following are available online at https://www.mdpi.com/2076-3921/9/5/402/s1, Table S1. Primers for RT-qPCR for genes and lncRNAs. Table S2. Summary of sequence read alignments to the reference genome; Table S3. Pearson correlation coefficients of duplicate bovine embryos of the same stage; Table S4. Normalized gene counts expressed in bovine embryos; Table S5. Differential expressed genes in untreated embryos between 8-16-cell and morula stage; Table S6. Differential expressed genes in GSH-treated embryos between 8-16-cell and morula stage; Table S7. Differential expressed genes between untreated and GSH-treated embryos at 8-16-cell stage; Table S8. Summary of LncRNAs expressed in bovine embryos; Table S9. Differential expressed lncRNAs in untreated and GSH-treated embryos developing from 8-16-cell stage to morula; Table S10. Co-expressed differentially expressed genes with 23 lncRNAs in untreated embryos; Table S11. Co-expressed differentially expressed genes with 23 lncRNAs in GSH treatment group; Table S12. Co-expressed differentially expressed genes with 36 lncRNAs in untreated embryos; Table S13. Co-expressed differentially expressed genes with 36 lncRNAs in GSH-treated embryos; Table S14. The protein-coding genes of 23 lncRNAs in bovine embryos; Table S15. The protein-coding genes of 36 lncRNAs in bovine embryos; Table S16. Quantitative real-time RT-PCR results of 10 genes and 8 LncRNAs between 8-16-cell stage embryos and morula treated with GSH; Table S17. Co-expressed differentially expressed genes with 14 lncRNAs participating in the glutathione metabolism pathway.
