# Supplementary material for: Long Non-Coding RNA and mRNA Profiling in Early-Stage Bovine Embryos Treated with Glutathione
Source: Antioxidants (Basel). 2020 May 8;9(5):402. doi: 10.3390/antiox9050402 (PMC7278749; doi:10.3390/antiox9050402)
Supplement: Supplementary file 1 [file antioxidants-09-00402-s001.zip › Table S15.docx]

**Table S15. Quantitative real-time RT-PCR results of 10 genes and 8 lncRNAs between 8-16-cell stage embryos and morula treated with GSH**

| Type | Genes Name | Log  (fold change) RNA-seq | Expression | Log  (fold change)  qRT-PCR |
| --- | --- | --- | --- | --- |
| Genes | *OOSP1* | -8.58 | Down | -2.97 |
|  | *THAP9* | -4.07 | Down | -2.05 |
|  | *ATP5L* | 3.45 | Up | 3.34 |
|  | *PSMA3* | 3.38 | Up | 3.55 |
|  | *UGP2* | 2.53 | Up | 1.47 |
|  | *RPS3A* | 3.86 | Up | 3.71 |
|  | *COX7A2* | 3.14 | Up | 2.71 |
|  | *MGST1* | 4.83 | Up | 3.52 |
|  | *IDH1* | 3.83 | Up | 5.36 |
|  | *RRM2* | 3.11 | Up | 3.17 |
| LncRNAs | CUFF.33095.2 | -22.10 | Down | -0.99 |
|  | CUFF.52291.1 | -21.09 | Down | -1.73 |
|  | CUFF.55358.1 | -1.65 | Down | -0.54 |
|  | CUFF.10166.1 | -6.04 | Down | -3.32 |
|  | CUFF.17837.1 | 3.62 | Up | 1.91 |
|  | CUFF.38204.3 | 6.06 | Up | 3.11 |
|  | CUFF.42178.2 | 1.23 | Up | 0.17 |
|  | CUFF.152963.1 | 2.78 | Up | 0.54 |

Fold change is expressed as the ratios of the values of the morula (n = 3) divided by those of the 8-16-cell embryos (n = 3).
