# Supplementary material for: Long Non-Coding RNA and mRNA Profiling in Early-Stage Bovine Embryos Treated with Glutathione
Source: Antioxidants (Basel). 2020 May 8;9(5):402. doi: 10.3390/antiox9050402 (PMC7278749; doi:10.3390/antiox9050402)
Supplement: Supplementary file 1 [file antioxidants-09-00402-s001.zip › Table S17.docx]

**Table S17. Primers for qRT-PCR for genes and lncRNAs**

| Genes | Primer sequence (5’- 3’) | Size (bp) |
| --- | --- | --- |
| *OOSP1* | F: TGACTGCCCTGTAACCTACG  R: ATCATTTGGCACACAGAGAGAA | 317 |
| *THAP9* | F: GCTGCCTCATTCTTCCATCCT  R: TGCTGCGTGAGAGATAGACC | 162 |
| *ATP5L* | F: GGAAAGACAGTGGGACACCT  R: GCGAGGTTACGGACAAACTG | 175 |
| *PSMA3* | F: GGCGTTGTCTTTGGGGTAGA  R: GCCAACAAACCTGCTACTGC | 116 |
| *UGP2* | F: CTCATTTGTGTCAGGGGTGC  R: AAGACAGAAACTTGGTGCGAC | 70 |
| *RPS3A* | F: TTGGGAAAACATTGGTCACGA  R: GGAGCACATTTTGTCACGGG | 206 |
| *COX7A2* | F: TACAGTTGGTGGAACGGCAT  R: CCAACTGATTGCTGGGAGGAT | 100 |
| *MGST1* | F: ACACGACGATTGTGCTTTCA  R: CGTTCCACCCTGTCATCTGT | 157 |
| *IDH1* | F:TGAAGAGCATGGCCGTAACTAA R:AGGAGCAGGAGACAAACACA | 170 |
| *RRM2* | F: ATTTTGCCACCATGCTCTCC  R: TTGGAAAGGTCCACCGGCT | 199 |
| Lnc RNAs | Primer sequence (5’- 3’) | Size (bp) |
| CUFF.33095.2 | F: CATGCAAGGAGTGAACCTGTG  R: GCCTTAGAGACAAGAGCAGGA | 91 |
| CUFF.52291.1 | F: TGCTGGTCTTCTTCCTTC  R: CATTCTTGGCGTTTCATC | 138 |
| CUFF.152963.1 | F: GCCTGAAGGTGAGGTTGC  R: GGTCTCCACTTAGCCCGTAG | 169 |
| CUFF.55358.1 | F: CTGTCTGGACGCAGTAGCC  R: GCAAAGCCTGAGAAGTGAGC | 200 |
| CUFF.17837.1 | F: TTCCGAGGTAGGGTAAGTGA  R: CGCCCAACTACATGATACAA | 194 |
| CUFF.38204.3 | F: AGACATGAGTGACGACGAG  R: ATACTGGAGTGGGTGGC | 377 |
| CUFF.42178.2 | F: AAAGCAGGAGGGTGGGTA  R: CAACGAACAGCAAGAGCC | 106 |
| CUFF.10166.1 | F: GAGGTTGGCAACTTTGATT  R: TAGCGTAGTGCTTTCTGATT | 363 |
